# Miscellany

**Published:** 1887-07

**Authors:** 


### MISCELLANY.

*Metaphysical Healing.*

It would scarcely seem worth while for the editor of the *Century Magazine* to devote seventeen pages of the valueable space of that periodical to combating the proposition that disease is purely a mental phenomenon. And yet there is one "college in Massachusetts, two in New York City, one in Brooklyn, four in Chicago and one in Milwaukee whose courses of instruction" are based upon that principle. The theory is that matter experiences no sensation, and that the physical body can no more be diseased in reality than a bar of iron can. Hence all human disease is in the mind, for the simple reason that it must be there or nowhere.

The Rev. Dr. Buckley thinks the spread of this doctrine of enough importance to be met seriously, and devotes himself accordingly to the task in the *Century*, as above mentioned. And he furnishes an interesting paper on the subject. The central point from which this nonsense radiates is the "Massachusetts Metaphysical College." It could not be called "medical," since medicine has no part in the "Science of Metaphysical Healing." It is so far above medicine that the latter is scarcely worth talking about. The course of instruction is short and quick. There is a "Primary class," each member of which pays $300 for twelve lessons ; a "Normal Class," which pays per pupil $200 for six lectures, and a "Metaphysical Obstetrics Class," each student paying $100 for six lectures.

The treatment is very simple. The sole seat of the disease being in the mind, the mind alone is to be doctored. Convince a person with rheumatism that the rheumatism is all in his mind, or metaphorically, all in his eye, and he can cast it out. If you sit down on a grassy bank and a bumble-bee you need not get up unless you think you must, for the sensation cannot be anywhere but in your mind; and if you keep it out of your mind, or, in other words, do not mind it, you are all right. Faith cure is an incidental branch of the science. Apparently a number of people —who would have got well anyhow— have been cured under this treatment and thus reinforced the delusion. The only grain of substance in it all is that there is a therapeutic value in a hopeful and cheerful state of mind in some cases.—*The World.*

---

*Deaths Among the German Philosophers.*

Death has been at work in Berlin among the German medical philosophers with great force this year. In the last few weeks two well-known and learned men have died whose names are not unknown in America. One is Wilhelm Hack, whose theory of the erectile tissue upon the turbinated bones created so much interest some years ago. The second is the pathologist, Carl Friedländer, a name connected with many valuable discoveries, but chiefly with a discovery bearing his name, the pneumococcus.—*Berlin letter in the Therapeutic Gazette.*

